# Pisa Syndrome in Parkinson's Disease: Evidence for Bilateral Vestibulospinal Dysfunction

**DOI:** 10.1155/2018/8673486

**Published:** 2018-10-15

**Authors:** Giulia Di Lazzaro, Tommaso Schirinzi, Maria Pia Giambrone, Roberta Di Mauro, Maria Giuseppina Palmieri, Camilla Rocchi, Michele Tinazzi, Nicola Biagio Mercuri, Stefano Di Girolamo, Antonio Pisani

**Affiliations:** ^1^Department of Systems Medicine, University of Rome Tor Vergata, Rome, Italy; ^2^Department of Otorhinolaryngology, University of Rome Tor Vergata, Rome, Italy; ^3^Department of Neuroscience, Biomedicine and Movement Sciences, University of Verona, Verona, Italy; ^4^Fondazione Santa Lucia IRCCS, Rome, Italy

## Abstract

**Introduction:**

Pisa syndrome (PS) is a postural complication of Parkinson's disease (PD). Yet, its pathophysiology remains unclear, although a multifactorial component is probable. Cervical vestibular evoked myogenic potentials (cVEMPs) explore vestibulospinal pathway, but they have not been measured yet in PD patients with PS (PDPS) to assess a potential vestibular impairment.

**Materials and Methods:**

We enrolled 15 PD patients, 15 PDPS patients, and 30 healthy controls (HCs). They underwent neurological examination and were examined with Unified Parkinson's Disease Rating Scale II-III (UPDRSII-III), audiovestibular workup, and cVEMP recordings. Data were analysed with Chi-square, one-way ANOVA, multinomial regression, nonparametric, and Spearman's tests.

**Results:**

cVEMPs were significantly impaired in both PD and PDPS compared with HCs. PDPS exhibited more severe cVEMP abnormalities with prevalent bilateral loss of potentials, compared with the PD group, in which a prevalent unilateral loss was instead observed. No clinical-neurophysiological correlations emerged.

**Conclusions:**

Differently from HC, cVEMPs are altered in PD. Severity of cVEMPs alterations increases from PD without PS to PDPS, suggesting an involvement of vestibulospinal pathway in the pathophysiology of PS. Our results provide evidence for a significant impairment of cVEMPs in PDPS patients and encourage further studies to test validity of cVEMPs as diagnostic and prognostic biomarkers of PD progression.

## 1. Introduction

Parkinson's disease (PD) is a common neurodegenerative disease characterized by both motor and nonmotor features. While motor signs (tremor, rigidity, and bradykinesia) mainly originate from the loss of dopaminergic neurons of *substantia nigra pars compacta* (SNpc) [1, 2], the pathophysiology of the large cohort of symptoms complicating the disease course, such as postural impairment, remains unclear, although it has been ascribed to the progressive degeneration of other cortical and subcortical structures [1].

Pisa syndrome (PS) is a peculiar postural complication, consisting in a persistent lateral trunk flexion (LTF) of more than 10° that can be reduced by passive mobilization or supine positioning, occurring throughout the disease course but favoured by the longer disease duration and responsible for severe disability [3–5]. Neurochemical imbalance in the basal ganglia homeostasis, defective sensory-motor integration, abnormal body scheme perception, together with a peripheral involvement consisting in myopathy, and other musculoskeletal affections have been proposed to contribute to the pathophysiology of PS [3, 6, 7]. However, the precise mechanisms have not been clarified yet.

Recently, a renewed interest emerged for vestibular evoked myogenic potentials (VEMPs), utilized for testing short-latency myogenic responses evoked by sound stimulation of vestibular system [8]. Specifically, cervical vestibular evoked myogenic potentials (cVEMPs) represent the sternocleidomastoid (SCM) muscle response to sound activation of saccule and signal transmission via the vestibulospinal tract. Thus, cVEMPs are a simple and reliable tool to explore the activity of the vestibulocollic reflex, linking the VIII and XI cranial nuclei, thereby representing an index of brainstem integrity [9–12]. Indeed, the vestibular system has been involved in the pathophysiology of PS [13], as well as in falls and other motor complications of advanced PD [13–15].

In spite of such evidence, to date, few studies addressed these issues in PD population. Evidence has been provided for the loss of muscular potentials, in the absence of clear correlations with relevant clinical features, such as disease duration and severity, gait, or sleep disturbances [15–17]. However, cVEMPs measurement has not been utilized yet to evaluate vestibular involvement in PS.

In this study, we recorded cVEMPs to assess vestibular integrity in PD patients with PS (PDPS) compared with both PD patients without PS and healthy controls (HCs).

## 2. Materials and Methods

### 2.1. Materials

A total of 60 subjects were consecutively recruited at the movement disorders outpatient service, Tor Vergata University Hospital, Rome, Italy, between 2015 and 2017. Subjects were divided into 3 groups: PD patients (*n*=15), PD patients with PS (PDPS, *n*=15), and healthy controls (HC, *n*=30). Idiopathic PD was diagnosed according to the British Parkinson's Disease Society Brain Bank criteria and confirmed by DaTscan [18]; PDPS patients showed trunk lateral flexion of at least 10°measured with a wall goniometer, alleviated by passive mobilization or supine positioning, according to recent diagnostic criteria [3]. HCs were healthy age-matched control subjects recruited from nonblood relatives of PD patients who did not show any neurological sign.

Exclusion criteria included dementia (MMSE score <24), cervical herniation, history of orthopaedic diseases (such as scoliosis), major spinal surgery, improper neck movements that could alter audiological assessment, middle ear diseases, hearing thresholds exceeding 50 dBHL, and medical therapy potentially interfering with vestibular function or potentially able to induce PS (e.g., neuroleptics).

All patients were evaluated by a movement disorder specialist, with Unified Parkinson's Disease Rating Scale (UPDRS) Sections II and III and Hoehn and Yahr scale (H & Y). Medical therapy was accurately recorded, and total levodopa equivalent daily dose (LEDD) was calculated for each patient.

All enrolled subjects underwent audiological workup to exclude hearing impairment, which could alter VEMPs recording. Then, cVEMPs were recorded, as described below. The entire assessment was performed in “on-therapy” state.

The study was conducted in agreement with ethical principles of Helsinki declaration. Informed consent was obtained from all participants.

### 2.2. Procedures

#### 2.2.1. cVEMPs Recording

The test was performed in a hypoechoic room, with the patient lying down on an examination bed, head positioned at 30°. Five surface electrodes were placed, the negative ones on the medium third of SCM muscles bilaterally, the positive ones in the centre of claviculae, and the ground electrode on the sternum. While maintaining tonic contraction of SCM, a bilateral acoustic stimulation (alternate click) was given to the subject continuously for 30 s, at a frequency of 4 Hz, duration of 100 *μ*s and at an intensity of 135 dB SPL, similarly in the three groups. The compound muscle action potentials (CMAPs) were then recorded as described [9].

**Figure 1:**
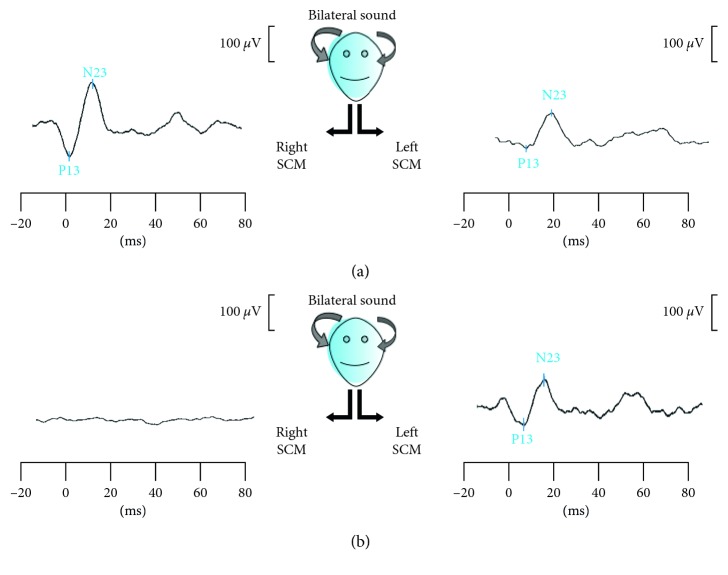
cVEMP recording in normal (a) and altered (b) conditions. In both (a) and (b) boxes, the two traces correspond, respectively, to right and left SCM muscle potential evoked by bilateral sound stimulation. P13 represents the potential's first peak of positivity, N23 the subsequent peak of negativity. In (a), cVEMPs are present bilaterally; in (b), cVEMP is lost on the right side.

Although muscle tonic activation was not measured, it was continuously monitored by visual feedback to assist the subject in maintaining the task. The recorded signals were amplified, averaged, and band-pass filtered (10–500 Hz). The procedure was performed at least twice to ensure reproducibility; grand average was analysed. CMAPs were evaluated qualitatively (present/absent) and quantitatively, measuring wave latencies and peak amplitudes from stimulus onset to the peak of the initial positivity (P13) and subsequent negativity (N23) [9]; interpeak latencies were also calculated [11] (Figure 1).

#### 2.2.2. Audiological Workup

Each subject underwent an otolaryngologic evaluation, including otoscopic examination, acoustic impedance test and pure-tone audiometry (PTA) to exclude middle ear disorders. Hearing loss was estimated bilaterally and for each pure-tone frequency stimulation (from 125 to 8000 Hz). The intensity threshold of the acoustic reflex was determined for each ear using 500 Hz, 1000 Hz, 2000 Hz, and 4000 Hz stimulus tones. The stimulus was presented either to the same ear as the compliance probe (ipsilateral reflex) or to the opposite ear (contralateral reflex).

For the Brainstem Auditory Evoked Responses (BAER) assessment, the active electrode was mounted to the middle of the forehead “Fpz,” the reference electrode to the ipsilateral mastoid “M1,” and the ground to the contralateral one “M2.” The test procedures followed the Sininger protocol [19]. Analysis of BAER was done quantitatively to assess the absolute latencies of waves I, III, and V and interpeak latencies of these waves (I–III, III–V, and I–V). Qualitative analysis of the waveform morphology included the subjective judgment on the shape and the quality of the waveforms.

### 2.3. Statistical Analysis

Differences among groups in categorical variables were calculated using the Chi-square test. Audiometric data were assessed separately by Kruskal–Wallis test for multiple comparisons and Wilcoxon test for repeated measures, as they were nonnormally distributed. The one-way ANOVA test with Bonferroni correction was performed for cVEMPs amplitude and latencies analysis among groups.

Correlations between neurophysiological parameters (unilateral or bilateral absence of cVEMPs, their amplitudes and latencies) and clinical features (disease duration, age of onset, UPDRS II-III as total scores and single subitems, degrees of LTF, and bending side) were assessed with Spearman's test. Moreover, after testing differences in cVEMPs alterations within the groups, the association between vestibular dysfunction and clinical presentation of PD was further assessed by means of multinomial logistic regression, using disease duration as covariate.

The level of statistical significance (*p* value) was set at 0.05. Statistical analyses were performed using the SPSS version 21 for Mac (SPSS Inc., Chicago).

## 3. Results

Main demographic, clinical, and neurophysiological data are summarized in Table 1.

Groups did not significantly differ in age, gender, and auditory testing (auditory thresholds and BAER latencies, data not shown). Disease duration, UPDRS part II-III scores, and LEDD were significantly higher in PDPS than in PD (respectively, *p* < 0.001, *p* < 0.001, *p* < 0.001, and *p*=0.013). Conversely, no difference emerged in the prevalence of dopaminergic medications used in PD and PDPS groups; specifically, 11/15 PD patients and 9/15 PDPS patients were on dopamine agonists, 10/15 PD and 9/15 PDPS were on MAO-B inhibitors, and 12/15 PD and 13/15 PDPS were on levodopa.

Consistent with previous findings, the main alteration we observed in cVEMPs within the PD groups was the unilateral or bilateral absence of evoked potentials (Figure 1); even if PDPS and PD patients had longer P13 and N23 latencies, no significant differences emerged among other analysed variables.

**Figure 2:**
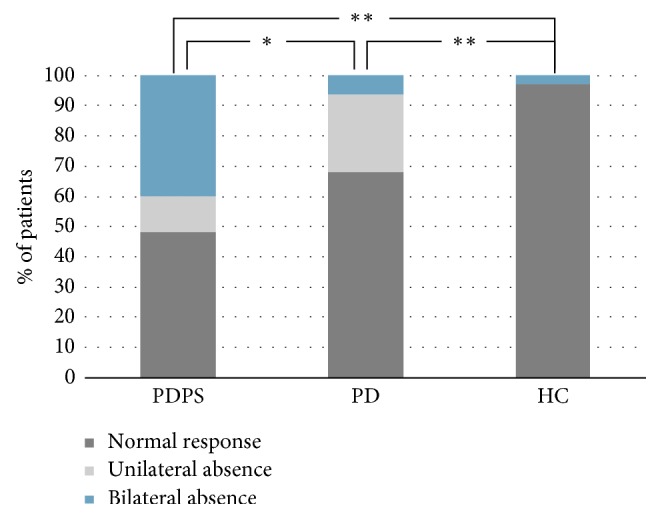
The plot summarizes the percentage of different cVEMPs responses in the three groups. Significance markers (^*∗*^ = *p* < 0.05; ^*∗∗*^ = *p* < 0.001) refer to the presence of “normal responses.” Other data are expressed in the text.

Table 1 shows, for each group, the percentage of “normal,” “unilateral absence,” and “bilateral absence” of cVEMPs. Chi-square analysis demonstrated that normal cVEMPs were significantly reduced in both PD and PDPS compared with HC (respectively, *p* < 0.001 and *p* < 0.001). However, the pattern of cVEMP abnormalities significantly differed between PDPS and PD (*p* < 0.0001), with PDPS showing lower normal responses (*p* < 0.05) and more frequent “bilateral absence” (*p* < 0.001); otherwise “unilateral absence” was more common in PD than in PDPS (*p* < 0.05) (Figure 2).

Multinomial logistic regression showed that “unilateral absence” of cVEMPs but not “bilateral absence” is significantly associated with pure PD condition (*T* = 21.9, *p* < 0.001), independently from disease duration. Moreover, either “unilateral” or “bilateral” cVEMPs absence are directly associated with PDPS condition (respectively, *T* = 3.4, *p*=0.02*p*=0.02 and *T* = 21.2, *p* < 0.001), as well as disease duration (*T* = 0.4, *p*=0.004).

Finally, correlation analysis between neurophysiological and clinical parameters did not provide any significant result among the groups.

## 4. Discussion

The neuropathological hallmark of PD is the ascending accumulation of *α*-synuclein immunoreactive Lewy bodies (LB) from SNpc and other brainstem structures to superior brain areas; however, LB deposition has been recently demonstrated in all brainstem fiber tracts and cranial nerve nuclei, including the vestibular system [20]. Of note, dopamine, whose levels are critically reduced in PD, is a fundamental modulator of vestibular nuclei function [21]. cVEMPs are now considered a reliable tool to assess vestibular and brainstem functions in distinct neurological disorders, including neuroaudiological, demyelinating and cerebrovascular diseases, migraine, and CNS tumours [8, 22]. Conversely, few studies examined the potential utilization of cVEMPs as a tool to monitor neurodegenerative diseases, particularly PD [15].

In our cross-sectional analysis of cVEMPs among PDPS, PD patients without PS and HCs without neurodegenerative conditions, we demonstrate that PD patients, compared with the latter group, have significantly abnormal vestibular evoked responses, consisting in unilateral or bilateral loss of cVEMPs. Furthermore, our findings indicate that the neurophysiological profile differs between PD patients with and without PS. The PD group mostly exhibits the unilateral absence of cVEMPs, whereas the PDPS group shows prevalently bilateral loss of responses. Our data on vestibular dysfunction may thus reflect the progression of brainstem neurodegeneration along disease course, with a progressive worsening in advanced stages, when postural complications are more frequently observed [23].

Despite the absence of significant correlations between neurophysiological parameters and specific clinical features, our results show a peculiar alteration of vestibular functioning in PS. While a previous study demonstrated unilateral peripheral vestibular hypofunction, ipsilateral to the LTF side [13], we observed a higher prevalence of bilateral alterations in PDPS patients here. A note of caution in the interpretation of our data is required, as our study lacks peripheral testing, which might have been useful in the anatomoclinical correlation. However, combining existing data with our novel observations, we hypothesize that central vestibular impairment might concur with peripheral dysfunction in patients with PS [13], suggesting the specific frailty of the vestibular system in this condition, even if longer disease duration might also play a role.

The study also included an audiometric examination of the three groups, which showed no significant differences in the hearing thresholds of subjects with PD, PDPS, and HCs. This finding partially disagrees with evidence by Vitale and colleagues [24, 25], instead demonstrating the occurrence of neurosensory hearing impairment in PD patients. The exclusion, in our study, of subjects with hearing thresholds over 50 dBHL even at a single frequency and additionally the smaller sample size and the different clinical-demographic features of the cohorts may account for this discrepancy.

## 5. Conclusions

cVEMPs are a noninvasive and inexpensive test exploring vestibulospinal tract, a crucial pathway for postural control. Despite the small sample size, here we observed that vestibulospinal dysfunction progresses in PD, with a milder involvement (unilateral loss of cVEMPs) in the absence of postural disabilities and more severe alterations (bilateral loss of cVEMPs) in the presence of PS. Therefore, we suggest that a major impairment of vestibular system might be involved in pathophysiology of PS, probably mirroring more advanced brainstem pathology in parallel with a longer disease duration. Further prospective studies on larger samples are mandatory to understand the associations between audiovestibular dysfunction and clinical progression of PD, allowing to establish the potential usefulness of cVEMPs as diagnostic and prognostic biomarkers for PD.

## Figures and Tables

**Table 1:** Clinical and electrophysiological data (y = years; M = males; F = females; mg = milligrams; ms = millisecond; mcV = microVolt; LTF = lateral trunk flexion; R = right; L = left; LEDD = levodopa equivalent daily dose; n.s. = statistically nonsignificant). Data are expressed as mean ± standard deviation (SD).

	PDPS (*n*=15)	PD (*n*=15)	HC (*n*=30)	
Age (y)	73.3 ± 3.6	69.6 ± 7.11	69.36 ± 6.67	n.s.
Gender (M/F)	8/7	7/8	15/15	n.s.
Disease duration (y)	8.7 ± 4.4	4.6 ± 3.83	—	*p* < 0.001
UPDRS II	12.4 ± 2.8	6.2 ± 3	—	*p* < 0.001
UPDRS III	30 ± 6.1	18.9 ± 6.1	—	*p* < 0.001
LTF (°)	21 ± 9	—	—	
Side of LTF (R/L)	7/8	—	—	
LEDD (mg)	814 ± 275	369 ± 202	—	*p*=0.013
Normal cVEMPs	48%	68%	97%	*p* < 0.001
Unilateral absence of cVEMPs	12%	26%	0%	*p* < 0.001
Bilateral absence of cVEMPs	40%	6%	3%	*p* < 0.001
P13 latencies (ms)	17.8 ± 10.82	14.32 ± 3.3	16.07 ± 8.97	n.s.
N23 latencies (ms)	26.53 ± 10.28	22.68 ± 4.2	25.77 ± 10.48	n.s.
P13 amplitudes (mcV)	46.18 ± 29.88	41.92 ± 37.75	40.57 ± 26.1	n.s.
N23 amplitudes (mcV)	−44.9 ± 35.26	−54.6 ± 34.01	−63.16 ± 42.31	n.s.
Interpeak latencies (ms)	8.6 ± 2.5	8.5 ± 2.3	9.6 ± 3.4	n.s.

## Data Availability

The data used to support the findings of this study are included within the article.
